# Intravascular Talcosis due to Intravenous Drug Use Is an Underrecognized Cause of Pulmonary Hypertension

**DOI:** 10.1155/2012/617531

**Published:** 2012-05-07

**Authors:** Christopher C. Griffith, Jay S. Raval, Larry Nichols

**Affiliations:** ^1^Department of Pathology, University of Pittsburgh Medical Center, Pittsburgh, PA 15213, USA; ^2^The Institute for Transfusion Medicine, Pittsburgh, PA 15220, USA

## Abstract

Intravenous injection of illegal drugs or medications meant for oral administration can cause granulomatous disease of the lung. This intravascular talcosis results in pulmonary fibrosis and pulmonary hypertension. Nine cases of histologically confirmed intravascular talcosis were reviewed with specific attention given to the clinical histories in these patients. Five autopsy cases were included in this series with detailed investigation in the anatomic features associated with intravascular talcosis and pulmonary hypertension. All nine patients showed perivascular and/or intravascular deposition of polarizable foreign material in their lungs. Intravascular talcosis as a result of previous intravenous drug use was not clinically suspected in any patient despite clinically diagnosed pulmonary hypertension in five. All patients showed dilatation of the right and left heart, but none had dilatation of the aortic valve. Congestive heart failure with hepatosplenomegaly was also common. We conclude that intravascular talcosis is an underdiagnosed cause of pulmonary hypertension in patients with known history of intravenous drug use.

## 1. Introduction

Pulmonary disease as a result of talc exposure has been well documented and can have multiple etiologies [1]. Inhalational talc exposure causes talc pneumoconiosis, while intravenous talc exposure causes intravascular talcosis. The disease symptoms and gross anatomic findings in these two different etiologies are essentially identical, and the histology of these two forms of talc-related lung diseases are also quite similar. Pulmonary deposition of insoluble microscopic foreign material results in a foreign body giant cell reaction within the lung parenchyma. Over time and continued exposure this process results in pulmonary fibrosis, in some cases extensively. The differentiating feature between these two diseases is the location of the foreign material. Inhalational talc pneumoconiosis results in an alveolar distribution, and intravascular talcosis leads to a perivascular pattern of deposition. In acute settings it is also possible to identify polarizable foreign material within intravascular spaces.

The source of foreign material in intravascular talcosis is through the intravenous injection of drugs. Illegal street drugs commonly contain an adulterant to increase the mass, and this adulterant commonly contains microscopic insoluble material. Another common source is the injection of prescription medications meant for oral use. In these medications which are ground for intravenous injection, there are fillers and binders added to the medications. In fact the term intravascular talcosis is a misnomer as talc is only one of several possible materials used as excipients that also include methylcellulose and crospovidone [2]. Special stains have been shown to have the ability to differentiate the composition of intravascular foreign material in diagnostically difficult situations [3].

With the intravenous injection of foreign material, the lungs represent the first capillary bed to serve as a filter to remove this material. Due to the size of much of this material it usually becomes lodged in the pulmonary vasculature. This results in acute small embolization of vessels. Over time the foreign material is deposited in perivascular tissues, and foreign body giant cell reaction occurs with associated fibrosis. This fibrosis of the lung parenchyma in turn results in the development of pulmonary hypertension in some patients. Intravascular talcosis as a cause of pulmonary hypertension has been well documented since the early description of this disease in the 1960s although the term intravascular talcosis has not been commonly used in the past [4, 5].

The diagnosis of intravascular talcosis has important social and medical treatment implications but is not commonly suspected in many patients even in the presence of known intravenous drug abuse [6]. Here we present nine cases of intravascular talcosis on biopsy and autopsy cases that were not clinically suspected. We give a detailed review of the patient histories. In five autopsy cases we also include detailed analysis of the pulmonary and cardiac disease relating to intravascular talcosis. The goal of this study is to increase awareness of intravascular talcosis as a cause for pulmonary hypertension and to present clinical and anatomic features that can suggest the diagnosis.

## 2. Materials and Methods

Cases of intravascular talcosis were identified through a natural language search for “talc” in our electronic laboratory information system over an eleven-year-period spanning 1/2000–12/2010. Surgical specimens and autopsy cases were included in the search. Final diagnoses were reviewed to select cases of intravascular talcosis, and slides were reviewed by the authors to confirm the diagnosis. Cases were excluded if slides or blocks were not available for review or the diagnosis was not confirmed. In autopsy cases, all slides were pulled and examined for the presence of polarizable foreign material in association with histocytic infiltration. The clinical histories of patients with confirmed intravascular talcosis were abstracted for relevant clinical data. This study was approved by the UPMC Institutional Review Board (IRB no. PRO11020060). 

### 2.1. Definition of Normal Metrics

Left ventricular hypertrophy was defined as wall thickness greater than 1.5 cm and right ventricular hypertrophy as wall thickness greater than 0.5 cm. Mitral valve dilation was defined as valve circumference greater than 9.9 cm in males and 9.1 cm in females, aortic valve dilation as valve circumference greater than 8.5 cm in males and 7.9 cm in females, tricuspid valve dilation as valve circumference greater than 11.8 cm in males and 11.1 cm in females, and pulmonic valve dilation as valve circumference greater than 7.5 cm in males and 7.4 cm in females [7]. Cardiomegaly was determined as a function of sex and body mass using the report by Kitzman et al. [7]. Splenomegaly was defined as spleen weight greater than 245 grams in males and greater than 190 grams in females. Hepatomegaly was defined as liver weight greater than 2000 grams in males and greater than 1800 grams in females. Increased lung weight was defined as combined lung weight greater than 1050 grams.

## 3. Results

### 3.1. Clinical Features of Intravascular Talcosis

A total of nine cases of intravascular talcosis are included in this study—five autopsy cases and four lung biopsy cases. Demographics for these patients are summarized in Table 1. The average age for all patients was 44 years. Three cases were diagnosed in patients with admitted drug use that was not recent, and these patients had a higher average age at 56 years. There was a predominance of males with only one female in the nine patients. 

The clinical histories of the patients were variable and are briefly described herein. Case 1 had a history of intravenous drug use and IgA nephropathy and was admitted for dyspnea and chest pain. Case 2 had a history of intravenous drug use and coronary artery disease and presented with acute chest pain. Case 3 had a history of remote intravenous drug use and history of stroke and was found unresponsive at his skilled nursing facility. Case 4 had a history of remote intravenous drug use, chronic obstructive pulmonary disease, and multiple pneumothoraces and was admitted for new pneumothorax and possible lung transplant evaluation. Case 5 had a history of intravenous drug use, chronic obstructive pulmonary disease, and chronic back pain requiring multiple surgeries and presented with a new episode of severe back pain. Case 6 had a history of remote intravenous drug use and right lung transplantation for talcosis and presented with increased work of breathing. Case 7 presented in the trauma suite with multiple traumatic penetrating chest injuries; a pneumonectomy was performed, but the patient did not survive. No history is available for this patient. Case 8 had a history of deteriorating lung function due to severe emphysema and presented for double lung transplantation. Case 9 had a history of multiple spine surgeries and presented with an enlarged periaortic lymph node and pulmonary infiltrate. While the majority of patients (6 of 8) with histologically confirmed intravascular talcosis had admitted intravenous drug abuse, two denied intravenous drug use. Other common clinical features were hepatitis C seropositivity (7 of 8 tested) and tobacco use (6 of 8 with history). 

Pulmonary hypertension was clinically diagnosed in five of the nine patients based on clinical features, cardiac catheterization, and echocardiography. Four patients had cardiac catheterization data available for review with all having elevated peak pulmonary artery pressures with an average of 43 mmHg (range 37–50 mmHg). End diastolic pulmonary artery pressures were increased in only two patients (average 15.25 mmHg, range 4–22 mmHg). Mean pulmonary artery pressures reported were elevated in two patients (average 25.25 mmHg, range 20–32 mmHg). Echocardiography (either transthoracic or transesophageal) studies were performed in six patients. Two patients had completely normal studies. Four patients had abnormal findings: mild right atrial dilatation (2), moderate-to-severe right ventricular dilatation (3), moderate pulmonary artery dilatation (2), moderate pulmonary hypertension (3; peak pulmonary systolic pressures 52–70 mmHg), moderate left atrial dilatation (2), mild left ventricular hypertrophy (1), severe left ventricular hypertrophy (1), and mildly decreased left ventricular ejection fraction (3). 

Five patients had chest computed tomography performed prior to pathological examination. Three showed emphysematous changes, one showed centrilobular and interstitial nodules, and one showed bibasilar atelectasis versus pneumonia. In one patient with severe panlobular emphysema, diffuse fibrosis was also evident. 

Pulmonary function testing was performed in four patients, and the following parameters were noted to be abnormal: forced vital capacity (FVC) (4 patients; 26–72% of predicted), forced expiratory volume in 1 second (FEV1) (4; 22–65% of predicted), diffusing capacity of the lung for carbon monoxide (DLCO) (4 patients; 1 with 62% of predicted and 3 others that were unattainable due to low lung volumes), and vital capacity (VC) (4 patients; 36 and 73% of predicted in 2 patients, and unattainable in 2 patients). 

Regarding relevant medical comorbidities, seven out of eight patients with known baseline status had a past medical history significant for hypertension that required medical management. Three patients had a history of chronic renal insufficiency (one of whom had IgA nephropathy and eventually required hemodialysis), and one patient developed acute renal failure prior to death. 

At the time of initial pathological diagnosis, no patients were clinically suspected of having intravascular talcosis. One patient had previously been diagnosed with inhalational talc pneumoconiosis at an outside institution based on biopsy. Following this diagnosis the patient had a single-sided lung transplant, and a later biopsy at our institution of the nontransplanted native lung was diagnostic of intravascular talcosis. This patient had a history of remote intravenous drug use. Another patient experienced multiple bilateral spontaneous pneumothoraces and was diagnosed with idiopathic pulmonary fibrosis; however, histological examination of the patient's lungs at autopsy demonstrated intravascular talcosis as a cause for his pulmonary fibrosis.

### 3.2. Histologic Features of Intravascular Talcosis

**Figure 1:**
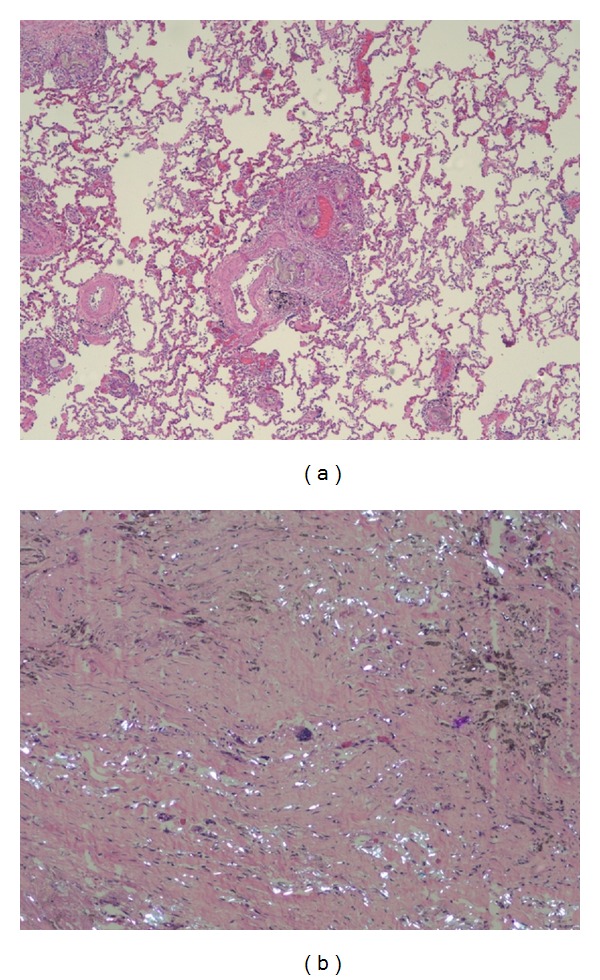
Low-power magnification of two cases of intravascular talcosis. The degree of fibrosis ranged from focal fibrosis in perivascular areas (a) to diffuse areas of fibrosis (b). Foreign material can be seen within areas of fibrosis associated with a foreign body giant cell reaction. Polarization of the material is evident in (b).

**Figure 2:**
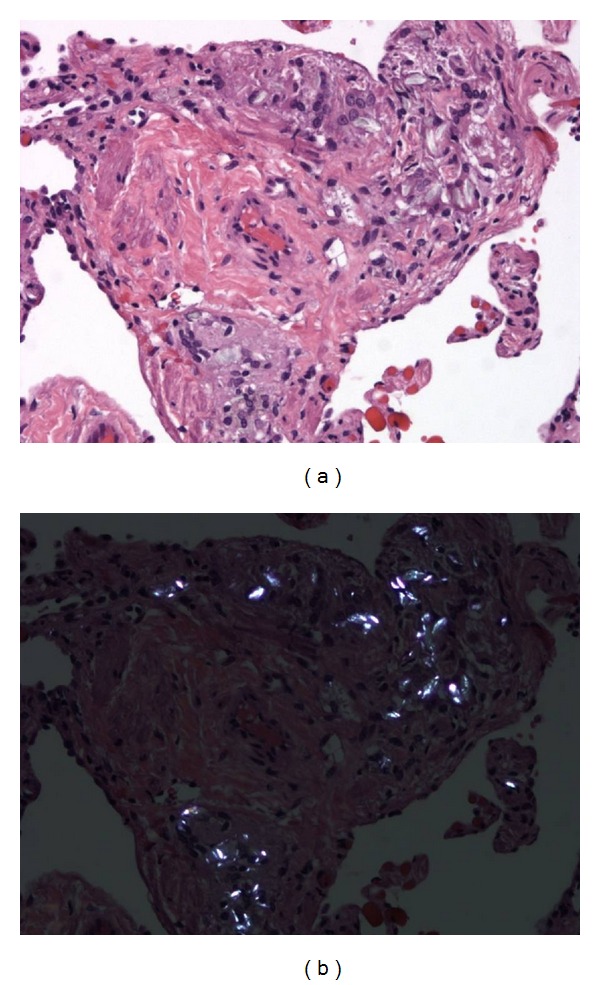
Higher magnification view of perivascular deposition of foreign material with foreign body giant cell reaction and fibrosis (a). The foreign material is highlighted under polarization (b).

**Figure 3:**
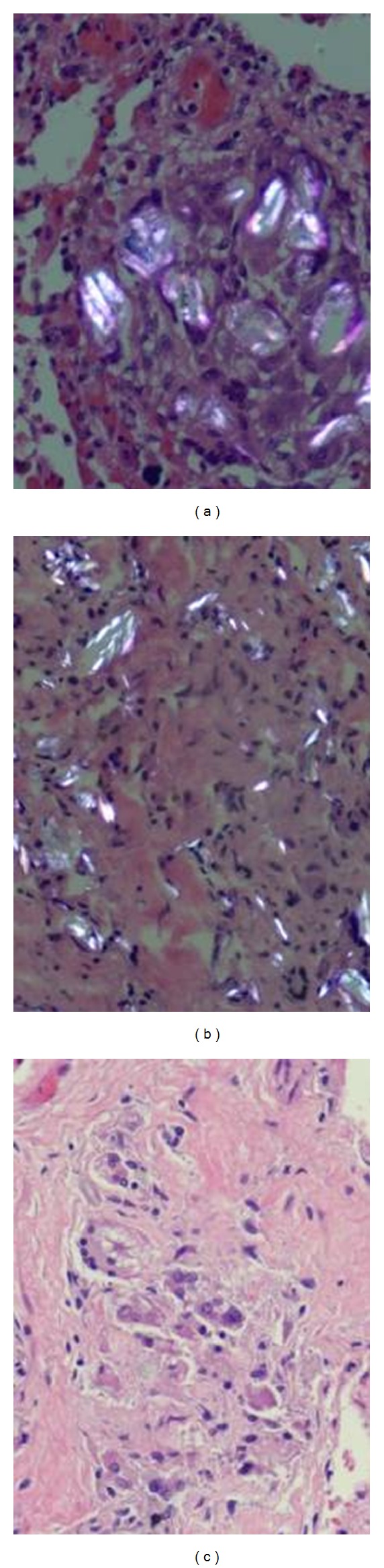
The foreign material deposits of intravascular talcosis have varying morphologies. Some cases showed more plate-like polarizable material (a) while other cases showed more needle-like morphology (b). Asteroid bodies are a common finding in intravascular talcosis (c).

The low power microscopic appearance in histologic sections of the lungs varied from focal areas of foreign body reaction and fibrosis to cases with extensive areas of fibrosis (Figure 1). The common finding in all cases was the diagnostic feature of a perivascular localization of foreign material deposition and fibrosis (Figure 2). In cases with extensive fibrosis the perivascular deposition could still be seen in areas with residual alveolated lung. The morphology of the foreign material itself varied with some showing more plate-like material and others more needle-like material (Figures 3(a) and 3(b)). Occasional asteroid bodies were found in some cases (Figure 3(c)). The histologic sections from autopsy cases and two of the four surgical pathology specimens showed some classical features associated with pulmonary hypertension [8]. Many medium caliber arteries showed hypertrophy of the muscular walls and larger arteries, when seen on sections, showed intimal proliferation similar to that seen in atherosclerosis. Many of the smaller caliber vessels failed to show these changes, and no plexiform vascular lesions were seen.

### 3.3. Anatomic Findings in Intravascular Talcosis Seen at Autopsy

**Figure 4:**
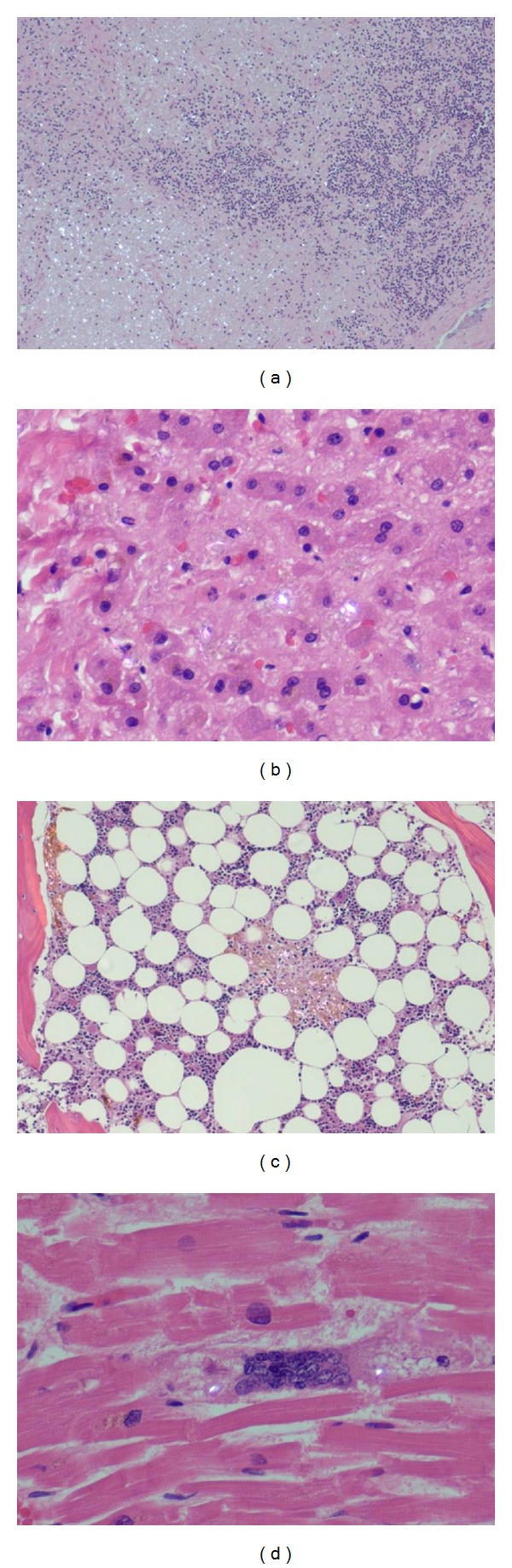
Systemic sites of foreign material are an important feature seen in intravascular talcosis. This material is smaller than seen in the lungs and only rarely incites a foreign body giant cell reaction. Representative figures show deposition in lymph node (a), liver (b), bone marrow (c), and heart (d).

Anatomic findings in the five patients having autopsy can be seen in Table 2. Causes of death included cardiac failure with pulmonary edema, cardiac arrhythmia secondary to pulmonary thromboembolus, multisystem organ failure secondary to sepsis, pulmonary fibrosis, and cardiopulmonary decompensation from pulmonary hypertension. All five patients had increased lung weights with an average of 1,894 grams. Cardiomegaly was present in three of five patients with an average heart weight of 600.4 grams. All five patients showed dilation of both right and left heart. The mitral and tricuspid valves were dilated in all 5 patients, average circumference of 10.9 cm and 13.5 cm, respectively. The pulmonic valve was dilated in 4 of 5 with an average circumference of 8.5 cm. None of the 5 patients had dilation of the aortic valve, average circumference 7.2 cm. Hepatomegaly was present in three of five and splenomegaly in four of five patients. The presence of polarizable foreign material was also present in extrapulmonary tissues in each of the five patients—bone marrow (3), lymph nodes (2), kidneys (2), spleen (2), liver (2), myocardium (2), thyroid (1), venous thrombus (1), and right ventricular thrombus (1) (Figure 4). One patient that is not deceased had prior biopsies of the liver and retina which showed similar polarizable foreign material. In each of the extrapulmonary tissues, the foreign material was intra-or perivascular, showed smaller particles than in the lungs, and lacked significant giant cell reaction. In one patient with transbronchial lung biopsy, previous biopsies of the retina and liver were reported to show polarizable foreign material. Four patients had evidence of moderate-to-severe coronary artery atherosclerosis, and three had histologic evidence of subendocardial and/or myometrial ischemia.

## 4. Discussion

The finding of perivascular or intravascular polarizable foreign material in the lungs is essentially diagnostic of intravascular talcosis due to intravenous injection of illegal drugs. The most important differential to establish is between intravascular talcosis and talc pneumoconiosis due to inhalation of microscopic dust. The differentiation of these two etiologies in foreign material associated granulomatous lung disease can be challenging as perivascular location in some ways includes the entire lung parenchyma. 

In this paper we searched for and selected cases of intravascular talcosis based on the histological location of the polarizable foreign material in perivascular or intravascular locations. Our findings show that none of the nine patients included in this study were clinically suspected of having intravascular talcosis due to intravenous drug abuse. This is despite the fact that five had clinically diagnosed pulmonary hypertension and six had admitted history of intravenous drug abuse. 

While the effects of intravascularly injected talc had an adverse impact on these patients' hemodynamic profiles as described in the current study, it is interesting to note that many of the histologic specimens demonstrated talc that was found to be distributed in the perivascular area and not intravascularly. While a definitive answer to explain this is elusive, one plausible hypothesis is that small talc particles are extravasated from the vascular space over time by the hydrostatic pressure within the vessels. The fact that a majority of patients in the current analysis had systemic hypertension would also facilitate this movement. With time, macrophages (and resulting giant cells) may also contribute to the movement of this foreign material farther from the intravascular space. Ultimately, fibrosis of this milieu of talc, immune cells, and perivascular stroma around the pulmonary vessels would, in addition to any remaining intravascular talc, contribute to the increased pulmonary artery pressures. 

Fibrosis and occlusion of small vessels within the lungs of these patients due to the intravascular and/or perivascular deposition of insoluble foreign material is the most probable cause of pulmonary hypertension in light of the clinical history and anatomic findings; however, contributions from other aspects of the patients' comorbidities, such as emphysema and fibrosis of other etiologies, cannot be completely excluded. The anatomic findings in the five patients undergoing autopsy were all likely related to the pulmonary hypertension in these patients. While the phenomenon of pulmonary hypertension in patients with intravenous drug use is well documented this is the first report to our knowledge to carefully examine the changes to the heart related to the disease. In the cases presented here all patients had dilatation of the left and right heart as determined by enlarged valve circumference. Cardiomegaly was also common being seen in three of five patients. Despite this finding none showed dilatation of the aortic valve. The reason for this is not known. Features of left sided congestive heart failure with hepatomegaly and splenomegaly were also common. 

The additional evidence to support the diagnosis in these patients was the common finding of fine birefringent material in systemic locations in these patients. This feature has been previously reported in patient with intravascular talcosis [9]. This finding should suggest the diagnosis of intravascular talcosis rather than talc pneumoconiosis in patients with pulmonary granulomas as inhalation of talc and related material should not result in systemic deposition. The smaller size of the material in the systemic sites compared to the lung parenchyma suggests that this material was small enough to escape the pulmonary capillary bed. 

Our analysis yielded findings from non-invasive testing which may increase suspicion for this disease in high risk individuals. Right-sided cardiac dilatation and pulmonary artery dilatation/hypertension were each found in at least half of patients that underwent echocardiography. Additionally, all patients (4/4) who underwent pulmonary function testing had decreased FVC and FEV1, along with either decreased or unattainable (due to low lung volumes) VC and DLCO. 

In the current study, hypertension requiring medical treatment was present in 7 out of 8 patients that were assessed. Only three patients had chronic kidney insufficiency (an additional patient had acute renal failure prior to death), and one of these required hemodialysis. Additionally, autopsy findings demonstrated either moderate-to-severe atherosclerotic disease or cardiac ischemia. These relevant cardiac and renal co-morbidities may have contributed to the observed cardiac findings, and while intravascular talcosis and its hemodynamic impacts may have exacerbated these conditions, their definitive relationship to intravascular talcosis is unknown. 

Intravascular talcosis as a result of intravenous injection of drugs is not an uncommon finding but is not universally seen in all intravenous drug users, and the finding of pulmonary hypertension has been previously reported in these patients [5]. The current study demonstrates that intravascular talcosis is an underrecognized cause of pulmonary hypertension despite the combination of pulmonary hypertension and a history of intravenous drug use in many of our patients. Patients with risk factors and findings identified herein may benefit from further clinical evaluation for intravascular talcosis.

## Figures and Tables

**Table 1:** Demographics for cases of talc granulomatosis.

Case	Specimen	Sex	Age (years)	Admitted IVDU	Hep C	Tobacco smoking	Pulmonary symptoms/diagnoses
1	Autopsy	M	37	Yes	Negative	no	Dyspnea Pulmonary HTN
2	Autopsy	M	40	Yes	Positive	yes	
3	Autopsy	M	62	Yes, remote	Positive	no	
4	Autopsy	M	55	Yes, remote	Positive	yes	IPF Pulmonary HTN Recurrent pneumothoraces
5	Autopsy	F	44	Yes	Positive	yes	COPD
6	Transbronchial biopsy	M	51	Yes, remote	Positive	yes	Dyspnea Pulmonary HTN s/p R lung transplant for talc exposure*
7	Pneumonectomy due to trauma	M	20	NA	NA	NA	
8	Double lung transplant native lungs	M	53	No	Positive	yes	Dyspnea Mild pulmonary HTN
9	Wedge resection	M	31	No	NA	yes	Dyspnea Pleuritic chest pain Pulmonary HTN

*Initially diagnosed with talc pneumoconiosis.

Abbreviations: HTN: hypertension, IPF: idiopathic pulmonary fibrosis, COPD: chronic obstructive pulmonary disease, s/p: status post, NA: not available.

**Table 2:** Anatomic features of intravascular talcosis.

Average organ weights (grams)		[range]
Lungs	(1894)	1490–2490
Heart	(600)	350–1030
Liver	(2421)	1300–4110
Spleen	(437)	80–860

Average heart valve circumference (cm)		

Mitral	(10.9)	10.5–11.5
Aortic	(7.2)	6.5–8
Tricuspid	(13.5)	12–15.5
Pulmonic	(8.5)	7–10
